# Anti-CD20 therapies in multiple sclerosis: From pathology to the clinic

**DOI:** 10.3389/fimmu.2023.1004795

**Published:** 2023-03-23

**Authors:** Jérôme de Sèze, Elisabeth Maillart, Antoine Gueguen, David A. Laplaud, Laure Michel, Eric Thouvenot, Hélène Zephir, Luc Zimmer, Damien Biotti, Roland Liblau

**Affiliations:** ^1^ Department of Neurology, Hôpital de Hautepierre, Clinical Investigation Center, Institut National de la Santé et de la Recherche Médicale (INSERM), Strasbourg, France; ^2^ Fédération de Médecine Translationelle, Institut National de la Santé et de la Recherche Médicale (INSERM), Strasbourg, France; ^3^ Department of Neurology, Pitié Salpêtrière Hospital, Paris, France; ^4^ Centre de Ressources et de Compétences Sclérose en Plaques, Paris, France; ^5^ Department of Neurology, Rothschild Ophthalmologic Foundation, Paris, France; ^6^ Department of Neurology, Centre Hospitalier Universitaire (CHU) Nantes, Nantes Université, Institut National de la Santé et de la Recherche Médicale (INSERM), Centre d’Investigation Clinique (CIC), Center for Research in Transplantation and Translational Immunology, UMR, UMR1064, Nantes, France; ^7^ Clinical Neuroscience Centre, CIC_P1414 Institut National de la Santé et de la Recherche Médicale (INSERM), Rennes University Hospital, Rennes University, Rennes, France; ^8^ Microenvironment, Cell Differentiation, Immunology and Cancer Unit, Institut National de la Santé et de la Recherche Médicale (INSERM), Rennes I University, French Blood Agency, Rennes, France; ^9^ Neurology Department, Rennes University Hospital, Rennes, France; ^10^ Department of Neurology, Centre Hospitalier Universitaire (CHU) Nîmes, University of Montpellier, Nîmes, France; ^11^ Institut de Génomique Fonctionnelle, UMR, Institut National de la Santé et de la Recherche Médicale (INSERM), University of Montpellier, Montpellier, France; ^12^ University of Lille, Institut National de la Santé et de la Recherche Médicale (INSERM) U1172, Centre Hospitalier Universitaire (CHU), Lille, France; ^13^ Université Claude Bernard Lyon 1, Hospices Civils de Lyon, Institut National de la Santé et de la Recherche Médicale (INSERM), CNRS, Lyon Neuroscience Research Center, Lyon, France; ^14^ Centre Ressources et Compétences Sclérose En Plaques (CRC-SEP) and Department of Neurology, Centre Hospitalier Universitaire (CHU) Toulouse Purpan – Hôpital Pierre-Paul Riquet, Toulouse, France; ^15^ Toulouse Institute for Infectious and Inflammatory Diseases (Infinity), University of Toulouse, CNRS, Institut National de la Santé et de la Recherche Médicale (INSERM), UPS, Toulouse, France; ^16^ Department of Immunology, Toulouse University Hospital, Toulouse, France

**Keywords:** multiple sclerosis, rituximab, ocrelizumab, ofatumumab, ublituximab, anti-CD20

## Abstract

The immune system plays a significant role in multiple sclerosis. While MS was historically thought to be T cell-mediated, multiple pieces of evidence now support the view that B cells are essential players in multiple sclerosis pathogenic processes. High-efficacy disease-modifying therapies that target the immune system have emerged over the past two decades. Anti-CD20 monoclonal antibodies selectively deplete CD20+ B and CD20+ T cells and efficiently suppress inflammatory disease activity. These monotherapies prevent relapses, reduce new or active magnetic resonance imaging brain lesions, and lessen disability progression in patients with relapsing multiple sclerosis. Rituximab, ocrelizumab, and ofatumumab are currently used in clinical practice, while phase III clinical trials for ublituximab have been recently completed. In this review, we compare the four anti-CD20 antibodies in terms of their mechanisms of action, routes of administration, immunological targets, and pharmacokinetic properties. A deeper understanding of the individual properties of these molecules in relation to their efficacy and safety profiles is critical for their use in clinical practice.

## 1 Introduction

Multiple sclerosis (MS) is a chronic immune-mediated disease of the central nervous system. The MS clinical course, neuroradiological manifestations, and response to therapy vary significantly among individuals (1, 2). Evidence over the past 10 years has shown that B cells play a key role in MS pathogenesis (3). B cells express the surface molecule CD20, that can serve as a specific target for monoclonal antibodies (mAbs). While rituximab was the first anti-CD20 therapeutic used in MS, ocrelizumab, a humanized anti-CD20 monoclonal antibody, was the first treatment approved for relapsing forms of MS (RMS) and primary progressive MS (PPMS) based on phase III positive outcomes (4, 5). Ofatumumab, a fully human anti-CD20 monoclonal antibody, represents the first subcutaneous (SC) self-administered anti-CD20 therapy approved for RMS (6). Finally, phase III clinical trials results for ublituximab in RMS patients were just recently published (7). In this review, we describe the neurological and immunological mechanisms involved in MS. We decipher differences and similarities between anti-CD20 mAbs. Finally, we discuss how these differences might influence efficacy and safety, as well as their relevance for clinical practice.

## 2 MS pathophysiology: Contribution of T and B cells

### 2.1 T cells in MS pathology

Evidence for immune system involvement in MS pathogenesis first came from the examination of active demyelinating lesions (8). These lesions show heterogeneity between patients but also vary according to the stage of the disease. Analysis of a wide collection of samples (51 biopsies and 32 autopsies) showed that infiltrating cells located in the demyelinating plaques mainly corresponded to macrophages and CD3+ T cells, despite the heterogeneity of the lesions. Plasma cells only accounted for a small fraction of the cells (8). Further analysis of T cells by flow cytometry revealed that CD8+ T cells outnumbered CD4+ T cells in active lesions of patients with MS (9). These CD8+ T cells preferentially expressed an effector memory and a tissue-resident phenotype (9–11). White matter lesions contained activated CD8+ T cells expressing a cytotoxic effector phenotype, and higher numbers of CD8+ T cells expressing co-inhibitory and co-stimulatory receptors (9).

Recent work using single-cell RNA sequencing compared gene expression of cerebrospinal fluid (CSF) cells from “MS-discordant monozygotic twin pairs” (12). “Healthy” co-twins had subclinical neuroinflammation with small magnetic resonance imaging (MRI) lesions. Clonally expanded CD8+ T cells showing activated tissue-resident memory T cell characteristics, plasmablasts, and CD4+ T cells were identified in both patients with MS and subjects with subclinical neuroinflammation. This suggested that there is early activation of adaptive immune cells in MS. Another study comparing patients with MS and healthy donors confirmed that clonally expanded CD8+ and CD4+ T cells in CSF expressed genes that are involved in T cell activation and cytotoxicity and were different from the T cell phenotype found in the blood (13). Several studies have further demonstrated that T cells derived from the blood of patients with MS displayed enhanced production of interferon (IFN)-γ, IL-17, and GM-CSF compared to healthy controls (14, 15). In addition, profiling of clones from CCR6+ myelin-reactive T cells, mainly corresponding to CD4+ T helper (Th17) cells from patients with MS, revealed increased production of proinflammatory cytokines (14). Interestingly, Th1-like Th17 effector memory cells have been found to be recruited into the CSF of patients with clinically isolated syndrome (CIS), which suggests an early activation of this cell population in MS (16). While the involvement of T cells in MS pathogenesis is no longer a matter of debate, recent profiling techniques have tremendously contributed to a deeper understanding of T cell subpopulations and their respective roles in MS development.

### 2.2 B cells in MS pathology

**Figure 1:**
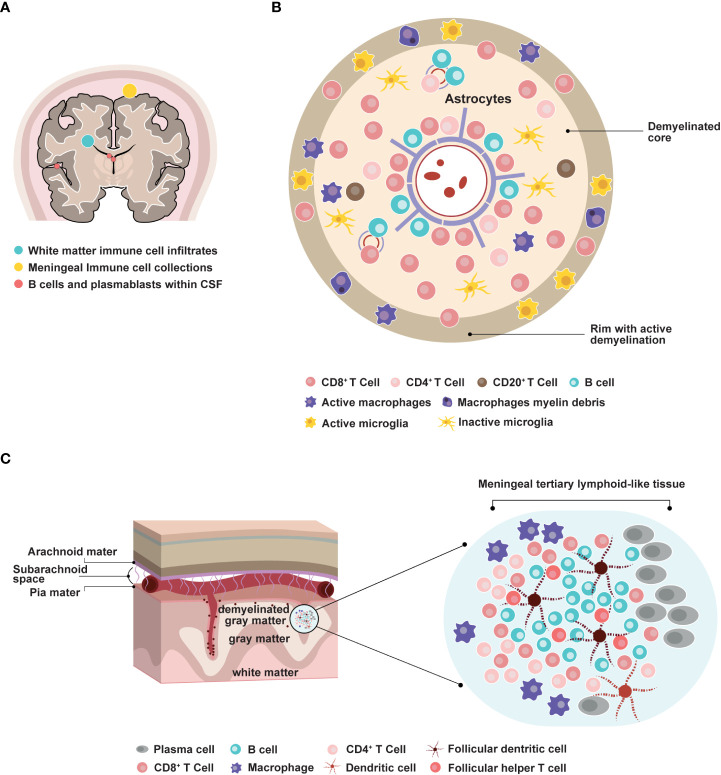
Involvement of B cells in multiple sclerosis pathophysiology. **(A)** Distribution of B cells in the central nervous system areas involved in MS pathology. CSF: cerebrospinal fluid. **(B)** Representation of an active MS lesion with a central inflamed vein, a demyelinated core, and a rim of active demyelination, and distribution of immune cell subpopulations (8, 9, 11, 17–20) **(C)** Representation of a meningeal follicle-like structure (17, 18, 21–25).

B cell contribution to MS pathology has emerged more recently. The analysis of autopsies at different disease stages indicated that inflammation in the brain occurred in RMS, secondary progressive MS (SPMS), and PPMS (11, 17). T and B cell infiltrates correlated with active lesions, where CD20+ B cells localized in the perivascular space of large veins. Plasma cell infiltrates were more abundant in the perivascular space and the meninges of patients with progressive MS (11, 17) (**Figures 1A–C**). Consistent with these findings, another study detected B cell aggregates resembling lymphoid follicle structures in the meninges of 20 out of 37 SPMS cases (21). These structures correlated with subpial demyelination, neuronal loss, and cortical atrophy. This indicated that B cell maturation and immune responses could occur locally within the CNS. Interestingly, deep sequencing of immunoglobulin G (IgG) heavy chain variable region genes in paired CSF and peripheral blood samples from patients with MS identified a small subset of clonally related B cells (26, 27). This suggested that B cell activation could take place in parallel in the periphery and within the CNS.

### 2.3 The potential role of Epstein-Barr virus in MS

Epstein–Barr virus (EBV), a highly B cell-tropic DNA herpes virus, induces asymptomatic infection in most people and can cause mononucleosis in adolescents and young adults (28).Analysis of postmortem brain tissue from patients with SPMS suggested that EBV infection could persist in B cells and plasma cells, especially within meningeal ectopic B cell follicles (29). The presence of EBV-infected cells was correlated with the activation of cytotoxic CD8+ T cells. Conversely, a recent single-cell RNA sequencing study did not detect EBV transcripts in CSF B cells in patients with RMS (30), and Angelini and colleagues reported that patients with active MS had a higher frequency of CD8+ T cells that were specific for lytic, but not latent EBV antigens when compared with patients with inactive MS or healthy donors (31). The authors postulated that EBV reactivation could occur during the active course of MS. This idea is further supported by a recent deep TCRβ repertoire analysis revealing that MS patients exhibited a higher number of TCRβ sequences against EBV (mostly lytic antigens) in peripheral blood T cells than matched healthy controls (32). A similar difference was found in MS-discordant monozygotic twins, with affected twins having greater EBV-specific TCRβ sequences than their healthy sibling.

To understand whether immune responses to EBV were detectable in patients with CIS and could predict conversion to MS, a cohort of 147 patients with CIS and 50 healthy controls were followed for seven years (33). The results indicated that patients with CIS had increased responses to EBV but not herpesvirus 6, measles, or cytomegalovirus. EBV-encoded nuclear antigen-1 (EBNA1) immune response was correlated with the number of T2 lesions at baseline, the number of T2 lesions, the presence of new T2 lesions, and the Expanded Disability Status Scale (EDSS) at follow-up. This suggested that EBNA1-specific IgG titers might be a prognostic marker for progression to MS. Interestingly, EBV viral reactivation was also increased in children with MS compared to healthy controls (34).

Assessment of antigen specificity of clonally expanded B cells from the CSF of patients with MS led to the identification of clones recognizing EBNA1 and a post-translationally modified form of GlialCAM, which is a self-antigen expressed by astrocytes and oligodendrocytes (26). This cross-reactivity was also detected in the serum of patients with MS and may provide a mechanistic link between EBV infection and MS pathogenesis.

Recent compelling results have implicated EBV as the trigger for MS development (35). In that large-scale study, the authors analyzed serum EBV antibodies from individuals who developed MS among a cohort of more than 10 million adults on active duty in the US military. The results indicated a 32-fold increase in the risk of developing MS after EBV infection. These findings warrant future efforts to elucidate how infection of B cells with EBV can initiate MS pathology.

### 2.4 Mechanisms underlying inflammation, demyelination, and neurodegeneration

Inflammation within the CNS leads to demyelination, subsequent neuronal loss, and axonal injury. Fundamental mechanisms driving demyelination and neurodegeneration include adaptive and innate immune systems, microglia activation, and oxidative damage (36). Analysis of 51 MS autopsies revealed that demyelination in the cerebral cortex was correlated with both inflammatory infiltrates in the meninges and oxidative-related degeneration of cortical neurons (18). By contrast, focal demyelinating lesions in the white matter occurred around blood vessels and involved retrograde neurodegeneration due to axonal loss. These observations suggested that local accumulation of inflammatory cells and production of soluble factors could induce demyelination and/or cytotoxic activity in a way that was directly or indirectly dependent on microglial activation (17, 18) (**Figure 1B**). Consistent with these results, *in vitro* experiments have indicated that lymphocyte-derived factors could influence the differentiation of oligodendrocyte precursor cells through crosstalk with microglial cells (37). Understanding these mechanisms *in vivo* is still challenging as it mostly relies on animal studies.

### 2.5 Role of peripheral B cells

B cells originate from hematopoietic stem cells in the bone marrow. Their maturation involves two distinct phases—antigen-independent maturation in the bone marrow and antigen-dependent maturation in the peripheral lymphoid tissue (38, 39). Pro-B cells (CD19- and CD20-) differentiate into pre-B cells (CD19+ CD20+), which develop into immature B cells expressing IgM while still in the bone marrow. Upon activation by an antigen and co-stimulatory factors, they evolve into mature B cells. In the germinal center, after Ig isotype switching, B cells become activated and exit to differentiate into memory B cells (CD27^low^), early plasmablasts (CD27^high^ and CD40L+), and ‘late’ plasmablasts (CD27+ CD38+). These cells migrate to the bone marrow, gut, spleen, tonsils, and brain under the direction of specific chemokines (CXCL12, CCL25, and CCL28), where they evolve into antibody-producing plasma cells. B cells can function as antigen-presenting cells. They can also produce proinflammatory cytokines, enhancing the inflammatory process (36). Various proposed mechanisms suggest that interactions between B and T cells drive MS pathogenesis (40). In particular, it has been postulated that peripheral B cells in MS escape the control of functionally impaired T regulatory cells. Activated B cells interact with Th cells in germinal centers and differentiate into memory B cells, which in turn induce Th effector cell activation. By expressing distinct chemokine receptors, adhesion molecules, and proinflammatory cytokines, highly pathogenic B and T cells break through the blood-brain barrier and become locally reactivated within the CNS, causing MS pathology.

## 3 Targets of anti-CD20 therapies

### 3.1 Structure of anti-CD20 monoclonal antibodies

Anti-CD20 mAbs target a cell membrane protein, “cluster of differentiation 20” (CD20), which is predicted to have four transmembrane helices with two extracellular loops. Several hypotheses have emerged regarding CD20 function – it has been suggested to function as an ion channel by some authors (41, 42), and to indirectly regulate calcium release from the B cell receptor by others (43). Structural analyses have revealed that CD20 can assemble as a compact dimeric double barrel (44). This challenged previous findings that it functions as an ion channel.

**Figure 2:**
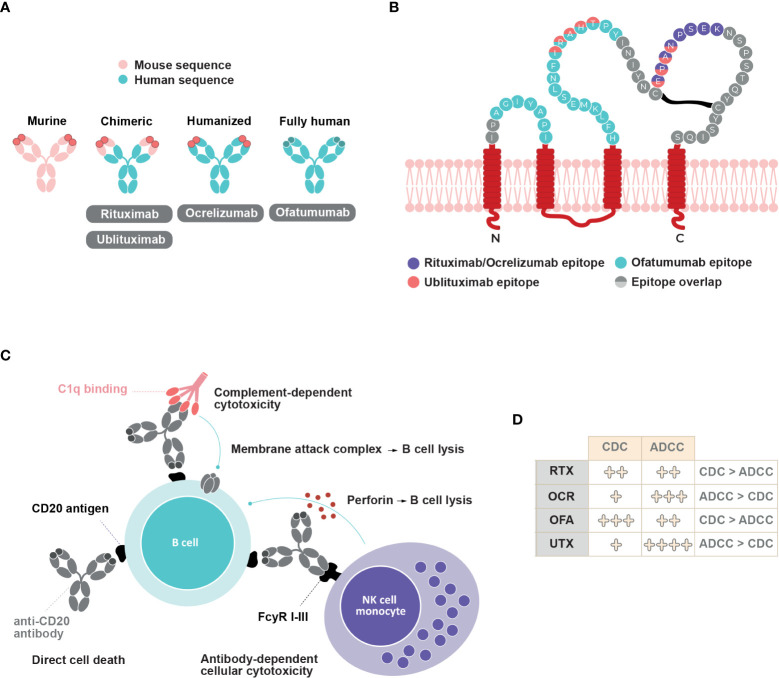
Anti-CD20 mAbs structure, epitope binding and mechanisms of action. **(A)** Structure of rituximab, ocrelizumab, ofatumumab and ublituximab antibodies **(B)** Binding epitopes of rituximab, ocrelizumab, ofatumumab and ublituximab on CD20. Adapted from Fox, 2021 (46) **(C)** Schematic representation of different mechanisms of action involved upon anti-CD20 mAbs binding and their relative contribution **(D)**. CDC, complement dependent cytotoxicity; ADCC, antibody-dependent cellular cytotoxicity; RTX, rituximab; OCR, ocrelizumab; OFA, ofatumumab; UTX, ublituximab.

Four anti-CD20 mAbs exist for MS treatment, as follows: rituximab, ocrelizumab, ofatumumab, and ublituximab. Although all anti-CD20 mAbs bind to the same target, they have distinct molecular and pharmacological features. Rituximab, an IgG1 mouse-human chimeric mAb, binds to amino-acid residues 168-175 on the large extracellular loop of CD20 (45). Rougé and colleagues also demonstrated that two rituximab Fabs could bind each CD20 dimer to form a circular rituximab-CD20 assembly that could allow complement recruitment (44). Ocrelizumab is a humanized glycosylated anti-CD20 IgG1 mAb that targets the large extracellular loop of CD20 on amino-acid residues 165-180, a different but overlapping epitope to that targeted by rituximab (45) (**Figures 2A, B**). Ofatumumab is the first fully human IgG1 mAb approved for the treatment of MS. Ofatumumab binds to discontinuous sequences of the small (amino-acid residues 74-80) and large (amino-acid residues 145-161) extracellular loops of CD20 (45) (**Figures 2A, B**). Ublituximab is an IgG1 chimeric mAb with a glycosylated Fc segment that enhances affinity for FcγRIIIa. It binds to amino-acid residues 158-159 and 168-171 on the large extracellular loop of CD20 (46, 47) (**Figures 2A, B**).

Immune responses generated against anti-CD20 mAbs may reduce therapy efficacy and/or cause adverse events. Murine-chimeric antibodies (rituximab and ublituximab) could potentially induce immunogenic responses compared to humanized (ocrelizumab) and fully human (ofatumumab) mAbs (45, 48).

### 3.2 Cellular targets of anti-CD20 mAbs

CD20 is expressed by pre-B cells in the bone marrow and naïve and memory B cells in the lymphoid tissues or germinal centers (39). Conversely, hematopoietic stem cells, most plasmablasts, and antibody-producing plasma cells do not express CD20. Although B cells represent the majority of CD20+ cells, a subset of CD3+ T cells also expresses CD20 on their cell surface (49, 50). These cells have been detected in the lymphatic tissues, blood, and CSF of healthy patients and were comparatively enriched in the blood and CSF of patients with MS. CD20+ T cells have a proinflammatory phenotype, and mostly correspond to CD8+ T cells with an effector memory T cell signature. CD20+ T cells in the CNS exhibit a tissue-resident memory phenotype (19). Further work is needed to understand the contribution of these cells to immune responses.

### 3.3 Mechanisms of action of anti-CD20 mAbs: *In vitro* evidence

Anti-CD20 mAbs molecular structure and epitope binding dictate the contribution of at least three distinct mechanisms of B cell depletion. The binding of anti-CD20 mAbs can induce direct cell death, complement-dependent cytotoxicity (CDC), and antibody-dependent cellular cytotoxicity (ADCC) (**Figures 2C, D**). ADCC involves antibody recognition by Fc-gamma receptors expressed on immune effectors such as natural killer cells and macrophages, which leads to direct cytotoxicity or phagocytosis (45, 51). Although all four anti-CD20 mAbs can induce the translocation of CD20 into lipid rafts, which allows for greater activation of complement proteins, the relative level of CDC activity varies between them. CDC activity predominates in rituximab and ofatumumab compared to ADCC activity (6, 45, 52). *In vitro* studies have indicated that ofatumumab exerts a two-fold greater ADCC activity than does rituximab, and a ten-fold higher CDC activity in rituximab-sensitive tumor cell lines (53, 54). Additionally, ofatumumab has a stronger binding affinity to the cell membrane and has a slower dissociation rate from CD20 than does rituximab (45, 55, 56). Ofatumumab induces greater CDC activity compared to ocrelizumab (77.1% *vs*. 7.1%) after a 2-hour exposure (57). In contrast, ADCC activity predominates over CDC activity in ocrelizumab and ublituximab (45, 46, 52). Compared with rituximab, ocrelizumab exhibits two- to five-fold greater ADCC activity and three- to five-fold lower CDC activity (58). Ublituximab, which is designed to have an enhanced affinity for FcγRIIIa, exerts greater ADCC activity than do rituximab, ocrelizumab, and ofatumumab (46, 59). Higher ADCC activity may allow lower doses and shorter infusion times (46), whereas weaker CDC activity may decrease infusion-related reactions (60).

### 3.4 Route of administration, dosing regimens, and kinetics of B cell depletion and reconstitution

Differences in the clinical efficacy and safety between anti-CD20 mAbs may depend on the route of administration. Patients treated with rituximab, ocrelizumab, and ublituximab receive intravenous (IV) infusion, whereas ofatumumab is delivered by SC self-injection (**Table 1**). IV-administered drugs offer fast bioavailability as they directly reach systemic blood circulation (61). In addition, they induce profound depletion of CD20-expressing B cells in the spleen due to the presence of loose capillaries (62). SC-administrated drugs are transported from the hypodermis to the lymphatics before entering the systemic circulation. Slower absorption can result in incomplete bioavailability compared to IV administration. Simultaneously, it may limit the side effects associated with high serum concentration (61).

**Table 1:** Administration route and dosing regimens of anti-CD20 mAbs in phase II/III clinical trials.

Administration route	Rituximab (OLYMPUS)	Rituximab (RIFUND-MS)	Ocrelizumab (OPERA I/II)	Ofatumumab (ASCLEPIOS I/II)	Ublituximab (ULTIMATE I/II)
	IV infusion	IV infusion	IV infusion	SC self-injection	IV infusion
Dosing regimen					
First dose	1 000 mg	1 000 mg	2 x 300 mg over ≥ 2.5 h	20 mg	150 mg over 4 h
Second dose and timing	1 000 mg at week 2		2 x 300 mg over ≥ 2.5 h at week 2	20 mg at weeks 1 and 2	450 mg over 1 h on day 15
Subsequent doses and timing	1 000 mg every 6 months	500 mg every 6 months	600 mg over ≥ 3.5 h or over ≥ 2 h every 6 months	Every month starting at week 4	450 mg over 1 h at weeks 24, 48 and 72

IV, intravenous; SC, subcutaneous.

Current dose regimens for rituximab, ocrelizumab, ofatumumab, and ublituximab result in rapid and near-complete depletion of circulating B cells, with varying rates of B cell reconstitution. Rituximab has been studied in two small clinical trials involving patients with RRMS (HERMES trial) (63) and with PPMS (OLYMPUS trial) (64), and more recently in a phase III clinical trial comparing rituximab and dimethyl fumarate in patients with RRMS (RIFUND-MS trial) (65). In the HERMES trial, patients received two 1000 mg IV infusions administered two weeks apart (at weeks 0 and 2) (63). In the OLYMPUS trial, patients were administrated two 1000 mg IV infusions at weeks 0 and 2, and subsequent two 1 000 mg infusions every six months at an interval of 14 days (64) (**Table 1**). Premedication was required before rituximab administration. Rituximab led to a > 95% reduction of CD19+ peripheral B cells within two weeks after the last infusion (63, 64). By week 48, the repletion of CD19+ cells corresponded to 30.7% of baseline values in the HERMES trial (63). In the OLYMPUS trial, peripheral B cells were above the lower limit of normal (LLN) in 35% of rituximab-treated patients 50 weeks after their last dose (64). In the RIFUND-MS trial, patients received a starting dose of 1000 mg IV rituximab followed by 500 mg every 6 months. No analysis of B cell depletion was reported (65).

Ocrelizumab efficacy and safety have been evaluated in the treatment of patients with RMS and PPMS, in the phase III trials OPERA I/II (4) and ORATORIO (5, 66), respectively. In both trials, patients treated with ocrelizumab received two 300 mg IV infusions (delivered over ≥ 2.5 hours) on days 1 and 15, and subsequent 600 mg infusions every six months (delivered over ≥ 3.5 hours) (**Table 1**). Premedication was recommended before ocrelizumab administration. Ocrelizumab induced an almost complete depletion of B cells by week 2 (after the first dose) in the OPERA I/II trials (4). For 90% of patients, the repletion of B cells occurred within 2.5 years after the last infusion (compared to the baseline or LLN), with a median time of 72 weeks (67).

Ofatumumab efficacy and safety have been studied in phase II MIRROR (68) and phase III ASCLEPIOS I/II trials in patients with RMS (6). Depletion of B cells occurred in a dose-dependent manner in the MIRROR trial (68). At week 12, B cell counts were between 2% and 25% of baseline levels. Repletion of B cells was also dependent on the dose, with 64-74% of patients reaching the LLN at week 132. Interestingly, B cell reconstitution started at approximately week 30 for the highest dose (60 mg every four weeks) and week 16-18 for the lower doses (3 mg, 30 mg, and 60 mg every 12 weeks), which suggests that the onset of B cell reconstitution was also dose dependent. Ofatumumab treatment consists of an SC self-administered injection of 20 mg once monthly (three initial doses administered weekly starting at week 0, followed by once-monthly doses starting at week 4, which allows rapid but safe B cell depletion with improved tolerability) and does not require premedication (6, 69) (**Table 1**). Rapid and near-complete depletion of B cells has been reported in 82–85% of patients by week 2 (6). By week 4, 94% of patients had less than 10 cells/μl. This percentage increased to 98% at week 12. B cell depletion was maintained independently of the patient’s body weight for as long as 120 weeks (69, 70). Clinical studies have shown a median time of 24.6 weeks for B cell reconstitution. This is consistent with PK-B cell modeling and simulation studies that estimated a median time of 23 weeks to B cell recovery (69).

Ublituximab has been evaluated in phase II (46, 71) and phase III clinical trials (7). In the phase III ULTIMATE I/II trials, patients with RMS received a 150 mg IV infusion (over 1-4 hours), followed by a 450 mg IV infusion (over 1 hour) 2 weeks later. Subsequent doses were administrated every 24 weeks up to week 96 (**Table 1**). Premedication was required before ublituximab infusion (7, 46). In phase II 48-week trial, B cell counts were significantly reduced 24 hours after the first dose (from 7.3% at baseline to 0.2%). A near-complete depletion of B cells (≥ 95%) occurred in all patients treated with ublituximab within two weeks after the second infusion. At week 48, no significant repletion was observed (46). In the phase III ULTIMATE I/II trials, CD19+ B cell depletion was achieved in 96% of patients treated with ublituximab 24 hours after the first dose (7). No significant increase in CD19+ was observed at the end of the trial, with cell counts still reduced by 97% in the ublituximab group.

### 3.5 Kinetics of B cell depletion and reconstitution in the CSF and tissues

Evaluating total B cell depletion is a challenge because blood counts do not necessarily reflect B cell depletion within tissues. An open-label phase II clinical trial analyzed B and T cells in the CSF of patients with RRMS treated with rituximab. The results indicated that a reduction in B and T cells in the CSF occurred in most patients 24 weeks after initial treatment (72). Blood infusion of rituximab allows minimal diffusion within the CNS through the blood-brain barrier (CSF:serum ratio 1:260), and its peak concentration in CSF remains very low (73). While anti-CD20 mAbs have proven efficacy for RMS treatment, they have failed to prevent long-term disability in SPMS. Pathophysiological data have suggested that meningeal ectopic lymphoid follicles are associated with SPMS (11, 17, 22). The proliferation of B cells and subpial inflammation have been linked to disease severity in SPMS, in that they lead to disease progression and neurodegeneration. This could in part, be explained by the inability of mAbs to cross the blood-brain barrier. The ongoing OBOE clinical trial (NCT02688985) evaluates biomarkers in the CSF of patients with RMS or PPMS who received ocrelizumab 600 mg every 24 weeks (74). The preliminary results have shown reductions in the concentration of neurofilament light chain, and the numbers of CD19+ B cells and CD3+ T cells, 12 and 24 weeks after ocrelizumab treatment. Interestingly, neurofilament light chain decrease over time was correlated with B and T cell numbers. This suggested a positive effect of ocrelizumab on reducing axonal injury. Analysis of B cells in the peripheral blood and CSF of a patient with RRMS treated with rituximab indicated a total depletion of CD19+ B cells in both compartments within eight weeks after rituximab administration (75). In this study, B cell depletion was still detected 6 months after the first infusion and was correlated with a reduction of gadolinium-enhancing T1 lesions.

To understand the kinetics of B cell depletion and recovery in different immune compartments, Häusler and colleagues used models of experimental autoimmune encephalomyelitis (76). The authors showed that ocrelizumab treatment reduced mature B cells in the bone marrow, blood, lymph nodes, and spleen. After treatment arrest, B cells simultaneously repopulated in the bone marrow and spleen before reappearing in blood. Ofatumumab treatment of cynomolgus monkeys induced a rapid and efficient depletion of B and CD20+ T cells but spared marginal zone B cells in the spleen and lymph nodes (77).

Analysis of B cell depletion and repletion in tissues remains a technical challenge, and many questions remain unanswered. In particular, the role and origin of plasma cells in the CNS of patients with MS remain unclear. Recent work examined the hypothesis that recirculating intestinal IgA-producing cells can regulate neuroinflammation (78). Data obtained in an experimental autoimmune encephalomyelitis mouse model suggested that some plasma cells found in the CNS originated from the gut and produced IgA, suppressing neuroinflammation *via* an IL-10-dependent mechanism. These intriguing findings warrant future efforts to understand the complexity of immune responses within MS tissues.

### 3.6 Subtypes changes and function

Anti-CD20 mAbs can quickly and efficiently deplete B cells in patients with MS. By contrast, B cells replenish at different times and frequencies according to the different antibodies. Several studies have examined how the absence and reoccurrence of B cells influence frequency, differentiation, and the activity of other immune populations, particularly T cells. A detailed analysis of peripheral B cell repletion after rituximab treatment for 24 months showed that reappearing B cells mainly corresponded to transitional and mature-naïve B cells, whereas memory B cell numbers were reduced (79). Interestingly, reappearing B cells corresponded to a more activated phenotype, as shown by enhanced expression of CD25. The cytokine B cell-activating factor of the TNF family (BAFF) is essential for B cell survival and differentiation. B cell depletion induced by rituximab increased BAFF production, a mechanism that could explain the survival of some B cells or the re-emergence of autoreactive B cells (80). Interestingly, genome-wide association studies have found that a variant in TNFSF13B (a disease risk allele), encoding BAFF, was associated with MS. This allele induced higher humoral immunity through increased levels of BAFF, B cells, and immunoglobulins (81).

While the involvement of EBV seroconversion on MS risk is getting clearer, the role of EBV infection and reactivation on the progression of established MS remains to be clarified. Since EBV mostly infects memory B cells, the observation of protracted reduced levels of memory B cells in the blood after B cell-depletion therapy (79) begs the question of whether changes in the proportion of EBV-infected B cells (or rates of latent *vs*. lytic infection) is one mechanism of action of anti-CD20 mAb therapy in MS. Dedicated longitudinal analysis of peripheral blood mononuclear cells biobanked prior to anti-CD20 mAb therapy, as well as before and after B cell repopulation in selected subgroups of treated MS patients should help tackle this important issue. These studies would interrogate both the B cell pool and the T cell repertoire (32).

**Figure 3:**
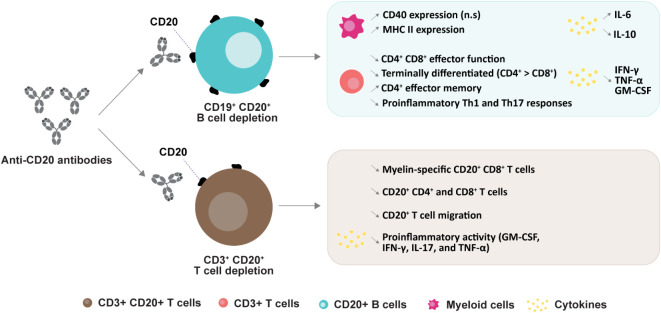
Anti-CD20 mediated changes in subtypes and their functions. CD20+ B cell depletion leads to an increase in CD40 expression (n.s: non-significant) and MHC II expression in myeloid cells (79); a decrease in CD4+ and CD8+ effector function (79), proinflammatory Th1 and Th17 responses (82), and numbers of terminally differentiated T cells, and an increase in CD4+ effector memory cells (85). Secretion of cytokines are changed upon B cell deletion (79, 85). CD20+ T cell depletion induces a decrease in myelin-specific CD20+ CD8+ cells (84), in both CD20+ CD4+ and CD8+ cells (86), in CD20+ T cell migration (85) and a decrease in pro-inflammatory cytokines (49, 87).

Anti-CD20 depletion can also result in a relative loss in CD4+ CD8+ T effector function, a decrease in terminally differentiated T cells (CD4+ > CD8+), and an increase of CD4+ effector memory T cells (79). B cell depletion with rituximab in patients with RRMS significantly reduced proliferation and proinflammatory cytokine (Th1 and Th17) responses of both CD4+ CD8+ T cells. B cell-mediated activation of T cells appeared to be associated, at least in part, with B cell production of the proinflammatory cytokines LT and TNFα (82). Another study investigated the role of B cells in “autoproliferation” or self-reactivity of peripheral Th1 cells (83). The authors showed that “autoproliferation” of Th1 cells was increased in patients carrying the HLA-DR15 haplotype (a genetic risk factor for MS) and was mediated by memory B cells in an HLA-DR-dependent manner. Depletion of B cells by anti-CD20 effectively reduced T cell autoproliferation. Although myelin proteins are considered to be potential autoantigenic targets, prior studies of myelin-reactive CD8+ T cells in MS were performed *in vitro*. In this study, *ex vivo* measurements of precursor frequencies and phenotypes of myelin-specific CD8+ T cells in the peripheral blood revealed an increased proportion of myelin-specific CD8+ T cells in patients with MS who exhibited a memory phenotype and expressed CD20 compared to control subjects (84). The proportion of memory and myelin-specific CD20+ CD8+ T cells was significantly reduced following anti-CD20 treatment (**Figure 3**).

Although B cells represent the primary target for anti-CD20 mAbs, a subpopulation of circulating CD3+ T cells also express CD20 (49, 50). Rituximab treatment in MS has been found to effectively target and deplete CD20+ CD3+ T cells in peripheral blood. Interestingly, this cell population replenished at earlier times and higher frequencies than CD20+ CD19+ B cells (49, 88). Similarly, it has been reported that depletion of CD3+ CD20+ T cells occurred two weeks after administration of 300 mg ocrelizumab (89). The APLIOS study also indicated a rapid depletion of specific CD20+ T cell subsets (CD20+ CD3+ CD8+ T cells) that are known to exhibit an activated phenotype (86) (**Figure 3**). Together, these findings suggest that depletion of CD20+ CD3+ T cells may contribute to some treatment effects.

## 4 Clinical and radiological efficacy of anti-CD20 mAbs

### 4.1 Rituximab

#### 4.1.1 Efficacy of rituximab in RRMS

##### 4.1.1.1 Phase II

Despite its broad use in clinical practice, rituximab is not approved for the treatment of RRMS, and few trials have analyzed its efficacy in MS. In the phase II HERMES trial, 69 patients with RRMS received 1000 mg of IV rituximab (*vs*. 35 patients who received a placebo) on days 1 and 15 (63) (**Table 2**). Total counts of gadolinium-enhancing lesions and new gadolinium-enhancing lesions were reduced at weeks 12, 16, 20, and 24 in patients who received rituximab compared to controls (p < 0.001). Rituximab-treated patients experienced a lower number of relapses at week 24 (14.5% *vs*. 34.3%, p = 0.02) and week 48 (20.3% *vs*. 40.0%, p = 0.04), compared to controls. The annualized relapse rate (ARR) was also decreased in patients who received rituximab at week 24 (p = 0.04), but not at week 48 (p = 0.08).

**Table 2:** Summary of clinical and MRI efficacy in phase II clinical trials.

	Rituximab	Ocrelizumab	Ofatumumab		Ublituximab
Trial Reference	HERMES Hauser et al., 2008	Kappos et al., 2011	Sorensen et al., 2014	MIRROR (Phase IIb) Bar-Or et al., 2018	Fox et al., 2021
MS form	RRMS	RRMS	RRMS	RRMS	RMS
Trial design	Randomized, double blind, placebo-controlled, multicenter	Randomized, parallel, double blind, dose finding study, multicenter	Randomized, double blind, placebo-controlled	Randomized, double blind, placebo-controlled, multicenter	Randomized, placebo-controlled, multicenter
Treatment groups	1000 mg IV infusions of rituximab or placebo on days 1 and 15	Ocrelizumab 600 mg 2 cycles (300 mg + 300 mg and 600 mg + 0 mg) Ocrelizumab 1000 mg (1000 mg + 1000 mg and 1000 mg + 0 mg) Placebo (only 1 cycle then ocrelizumab 600mg) Interferon beta-1a (only 1 cycle 30μg per week then ocrelizumab 600 mg)	2 IV infusions of either ofatumumab (100, 300, or 700 mg) or placebo 2 weeks apart	Placebo or subcutaneous ofatumumab 3 mg, 30 mg, or 60 mg every 12 weeks or 60 mg every 4 weeks	Ublituximab 150 mg on day 1 (1-4 hours) and then 450 or 600 mg on day 15 and week 24 (1-3 hours)
Patients (ratio)	n=104 (2:1)	n=220 (1:1:1:1)	n=38 (2:1)	n=232 (2:1:1:1:2)	n=48 (3:1)
Follow-up	48 weeks	96 weeks	Two 24-week treatment periods	24 weeks	48 weeks
Primary objective	Number of gadolinium-enhancing lesions T1-weighted MRI brain scans at weeks 12, 16, 20, and 24	Total number of gadolinium-enhancing T1 lesions observed on brain MRI scans at weeks 12, 16, 20, and 24	Cumulative number of new gadolinium-enhancing lesions, T2 lesions, and T1 hypointense lesions measured on monthly MRI.	Cumulative number of new gadolinium-enhancing lesions at week 12 (based on T1-weighted MRI scans at weeks 4, 8, and 12)	Responder rate, defined as the proportion of ublituximab-treated patients with ≧̸95% peripheral CD19+ B-cell depletion from baseline within 2 weeks after the second ublituximab infusion
ARR (mean, 90% or 95% CI)	Between week 0 and 24 weeks rituximab vs placebo: 0.3 vs. 0.8, p=0.04 Between 0 and 48 weeks rituximab vs placebo: 0.4 vs. 0.7, p=0.08	Annualized relapse rate by week 24: Ocrelizumab 600 mg: 0.13 (0.53-0.29); p=0.0005 versus placebo; p=0.03 versus interferon beta-1a Ocrelizumab 2000 mg: 0.17 (0.05-0.3); p=0.0014 versus placebo; p=0.09 versus interferon beta-1a Placebo: 0.64 (0.43-0.94) Interferon beta-1a: 0.36 (0.22-0.60)	n/a	n/a	ARR at baseline: 1.45 ARR at week 48: 0.07
Gadolinium-enhancing T1 lesions (mean;SD or 95% CI)	Mean number of lesions at weeks 12, 16, 20, and 24: Rituximab: 0.5 ± 2.0 as Placebo: 5.5 ± 15.0 (p<0.001) Mean number of new lesions Rituximab: 0.2 ± 0.4 Placebo: 4.5 ± 12.6 (p<0.001)	Total number of galodinium-enhancing T1 lesions over weeks 12, 16, 20, and 24: Ocrelizumab 600 mg: 0.6; p<0.0001 versus placebo or interferon beta-1a Ocrelizumab 2000 mg: 0.2; p<0.0001 versus placebo or interferon beta-1a Placebo: 5.5 (12.5) Interferon beta-1a: 6.9 (16.0)	For weeks 0 –24 New T1 GdE lesions (p< 0.001) ofatumumab (-4.0) versus Placebo (-1.0), Total number of T1 GdE lesions (p< 0.001), ofatumumab (= -6.0) versus Placebo (= 0.0)	65% reduction of cumulative Gd-enhancing lesions for all ofatumumab groups between weeks 0 and 12 (rate ratio 0.35, 95% CI 0.221–0.548, p < 0.001)	Mean number at baseline: 3.63 ( ± 7.80). At weeks 24 and 48, no new or persisting lesion (100% reduction from baseline; p=0.003).
MRI T2 lesions (mean; SD or 95% CI)	Changes in lesions volume At week 24: rituximab: −163.1 ± 1187.6 placebo: 436.3 ± 1358.4 (p=0.008) At week 36: rituximab: −175.4 ± 1188.1 placebo: 417.8 ± 1305.1 (p=0.004)	Change in volume of T2 lesions: Ocrelizumab 600 mg: -841.4 (2702.2); p=0.2 versus placebo Ocrelizumab 2000 mg: -578.1 (2109.2); p=0.2 versus placebo Placebo: -114.0 (1400.8) Interferon beta-1a: 996.7 (4418.1) Total number of new or enlarging T2 lesions at week 24: Ocrelizumab 600 mg: 0.0 (0.1); p<0.0001 versus placebo Ocrelizumab 2000 mg: 0.0 (0.1); p<0.0001 vs placebo Placebo: 1.4 (3.3) Interferon beta-1a: 1.8 (5.2)	New and/or enlarging T2 lesions (p< 0.001) ofatumumab (-4.0) versus placebo (0.00).	n/a	Mean T2 lesion volume at baseline: 15,410 mm^3^ 7.3% decrease by week 24 from baseline (p=0.006) 3.6% decrease between week 24 and week 48 (p=0.019) Total decrease from baseline of 10.6% (p=0.002) New or enlarging T2 lesions from baseline to week 24: 0.20 ( ± 0.43) New or enlarging T2 lesions from week 24 to week 48: 0.04 ( ± 0.29)

n/a, not applicable.

##### 4.1.1.2 Phase III

The RIFUND-MS trial evaluated the efficacy and safety of rituximab compared to dimethyl fumarate in patients with RRMS or CIS (65). In this study, 100 patients received 1 000 mg of IV rituximab followed by 500 mg every 6 months and 100 patients received oral dimethyl fumarate 240 mg twice daily for an observation period of 24 months. A total of five patients were removed from the intention-to-treat analysis. Relapses occurred in 3% of patients treated with rituximab *vs*. 16% of patients in the dimethyl fumarate group, corresponding to a risk ratio of 0.19 (p=0.0060) (**Table 3**). The annualized relapse rate was inferior in the rituximab group (0.015) compared to the dimethyl fumarate group (0.087). Secondary outcomes indicated that no new MRI activity was detected in 79% and 63% of patients treated with rituximab and dimethyl fumarate, respectively. Patients treated with rituximab had reduced numbers of T2 lesions (mean: 0.3 *vs*. 1.5, p=0.0047) and Gd+ lesions (mean: 0.04 *vs*. 0.26, p=0.0062) compared to patients who received dimethyl fumarate. No significant difference was observed between the groups for the Expanded Disability Status Scale score (**Table 3**).

**Table 3:** Summary of clinical and MRI efficacy in phase III clinical trials.

	Rituximab	Rituximab	Ocrelizumab			Ofatumumab		Ublituximab	
**Trial** **Reference**	OLYMPUS Hawker et al., 2009	RIFUND-MS Svenningsson et al., 2022	OPERA I Hauser et al., 2017	OPERA II Hauser et al., 2017	ORATORIO Montalban et al., 2017	ASCLEPIOS I Hauser et al., 2020	ASCLEPIOS II Hauser et al., 2020	ULTIMATE I Steinman et al., 2022	ULTIMATE II Steinman et al., 2022
**MS form**	PPMS	RRMS / CIS	RMS	RMS	PPMS	RMS	RMS	RMS	RMS
**Trial design**	Multicenter, randomized, double-blind, placebo-controlled trial	Multicenter, randomized rater-blinded, active-comparator,	Multicenter, randomized, double-blind, double-dummy, active-controlled, parallel-group trial	Multicenter, randomized, double-blind, double-dummy, active-controlled, parallel-group trial	Multicenter, randomized, parallel-group, double-blind, placebo-controlled trial	Multicenter, randomized, double-blind, double-dummy, active-controlled trial	Multicenter, randomized, double-blind, double-dummy, active-controlled trial	Multicenter, randomized, double-bind, double-dummy, active-controlled trial	Multicenter, randomized, double-bind, double-dummy, active-controlled trial
**Treatment groups**	2 x 1000 mg intravenous infusions of rituximab in the interval of 2 weeks throughout the whole study Placebo	Single 1000 mg intravenous infusion of rituximab, followed by 500 mg every 6 months Dimethyl fumarate 240 mg twice daily	Ocrelizumab 600 mg per 24 weeks Interferon beta-1a 44 µg three times per week	Ocrelizumab 600 mg per 24 weeks Interferon beta-1a 44 µg three times per week	Ocrelizumab 600 mg per 24 weeks Placebo	Ofatumumab at a dose of 20 mg subcutaneously every 4 weeks after 20-mg loading doses at days 1, 7, and 14 and oral teriflunomide at a dose of 14 mg once daily vs placebo	Ofatumumab at a dose of 20 mg subcutaneously every 4 weeks after 20-mg loading doses at days 1, 7, and 14 and oral teriflunomide at a dose of 14 mg once daily vs placebo	Intravenous ublituximab at a dose of 150 mg on day 1, followed by 450 mg on day 15 and at weeks 24, 48, and 72 Teriflunomide at a dose of 14 mg once daily	Intravenous ublituximab at a dose of 150 mg on day 1, followed by 450 mg on day 15 and at weeks 24, 48, and 72 Teriflunomide at a dose of 14 mg once daily
**Patients (ratio)**	N=439 (2:1)	N=200 (2 :1)	N=821 (1:1)	N=835 (1:1)	N=732 (2:1)	N=927 (1:1)	N=955 (1:1)	N=549 (1:1)	N=545 (1:1)
**Follow-up**	96 weeks	24 months	96 weeks	96 weeks	120 weeks	up to 30 months	up to 30 months	96 weeks	96 weeks
**Primary objective**	Time to CDP, a prespecified increase in EDSS sustained for 12 weeks	Proportion of patients with at least one relapse	Annualized relapse rate (ARR) at 96 weeks	Annualized relapse rate (ARR) at 96 weeks	Percentage of patients with disability progression confirmed at 12 weeks in a time-to-event analysis	Annualized relapse rate (ARR) up to the end of the trial	Annualized relapse rate (ARR) up to the end of the trial	Annualized relapse rate (ARR) by week 96	Annualized relapse rate (ARR) by week 96
**ARR (mean, 90% or 95% CI)**	n/a	Relapses had occurred in 3% of patients in the rituximab group and 16% of patients in the dimethyl fumarate group (risk ratio: 0·19 (95% CI 0·06–0·62; p=0·0060) ARR: 0.015 with rituximab and 0.087 with dimethyl fumarate	ARR: 0.16 with ocrelizumab and 0.29 with interferon beta-1a (rate ratio: 0.54 (0.40 to 0.72); p<0.001)	ARR: 0.16 with ocrelizumab and 0.29 with interferon beta-1a (rate ratio: 0.53 (0.40 to 0.71); p<0.001)	n/a	ARR: 0.11 with ofatumumab and 0.22 with teriflunomide (difference, −0.11; 95% confidence interval [CI], −0.16 to −0.06; p<0.001)	ARR: 0.10 with ofatumumab and 0.25 with teriflunomide (difference, −0.15; 95% CI, −0.20 to −0.09; p<0.001)	ARR: 0.08 with ublituximab and 0.19 with teriflunomide (rate ratio, 0.41; 95% CI, 0.27 to 0.62; p<0.001)	ARR: 0.09 with ublituximab and 0.18 with teriflunomide (rate ratio, 0.51; 95% CI, 0.33 to 0.78; p = 0.002)
**Gadolinium-enhancing T1 lesions (mean; SD or 95% CI)**	n/a	Rituximab: 0.04 (0.20) Dimethyl fumarate: 0.26 (0.70) p=0·0062	Ocrelizumab: 0.02 (0.01-0.03) Placebo: 0.29 (0.20-0.41) Rate ratio: 0.06 (0.03-0.10); p<0.001	Ocrelizumab: 0.02 (0.01-0.04) Placebo: 0.42 (0.31-0.56) Rate ratio: 0.05 (0.03-0.09); p<0.001	n/a	Ofatumumab: 0.01 (0.01 to 0.02) Teriflunomide: 0.45 (0.36 to 0.58) Rate ratio: 0.03 (0.01 to 0.05); p<0.001	Ofatumumab: 0.03 (0.02 to 0.05) Teriflunomide: 0.51 (0.40 to 0.66) Rate ratio: 0.06 (0.04 to 0.10); p<0.001	Ublituximab: 0.02 (0.01 to 0.03) Teriflunomide: 0.49 (0.35 to 0.68) Rate ratio: 0.03 (0.02 to 0.06) p<0.001	Ublituximab: 0.01 (0.00 to 0.02) Teriflunomide: 0.25 (0.16 to 0.39) Rate ratio: 0.04 (0.02 to 0.06) p<0.001
**MRI T2 lesions (mean; SD or 95% CI)**	T2 volume change from baseline to week 96 rituximab: 1,507 (3739) placebo: 2,205 (4306) p<0.001	Rituximab: 0.3 (0.7) Dimethyl fumarate: 1.5 (4.0) p=0·0047	Total number of new or newly enlarged hyperintense lesions on T2-weighted MRI by week 96: Ocrelizumab: 0.32 (0.26-0.41) Placebo: 1.41 (1.12-1.78) Rate ratio: 0.23 (0.17-0.30); p<0.001)	Total number of new or newly enlarged hyperintense lesions on T2-weighted MRI by week 96: Ocrelizumab: 0.33 (0.26-0.41) Placebo: 1.90 (1.54-2.36) Rate ratio: 0.17 (0.13-0.23); p<0.001	Adjusted geometric mean percent change in total volume of lesions on T2-weighted images from baseline to week 120: Ocrelizumab: –3.37 (–4.99 to –1.72) Placebo: 7.43 (4.97 to 9.94) HR: 0.90 (0.88-0.92); p<0.001	Mean number of new or enlarging lesions per year: Ofatumumab: 0.72 (0.61 to 0.85) Teriflunomide: 4.00 (3.47 to 4.61) Rate ratio: 0.18 (0.15 to 0.22) p<0.001)	Mean number of new or enlarging lesions per year: Ofatumumab: 0.64 (0.55 to 0.75) Teriflunomide: 4.15 (3.64 to 4.74) Rate ratio: 0.18 (0.15 to 0.22) p<0.001	New or enlarging lesions: 0.21 (0.14 to 0.32) with ublituximab and 2.79 (2.14 to 3.64) with teriflunomide group Rate ratio: 0.08; 95% CI, 0.06 to 0.10; p<0.001)	New or enlarging lesions: 0.28 (0.20 to 0.40) with ublituximab and 2.83 (2.13 to 3.77) with teriflunomide Rate ratio: 0.10; 95% CI, 0.07 to 0.14; p<0.001
**CDP (%; HR; 95% CI)**	No evidence of significant difference in time to CDP between the rituximab and placebo groups (p=0.14). Week 96 CDP rates were 38.5% for the placebo and 30.2% for the rituximab group	n/a	Disability progression confirmed at 24 week: Ocrelizumab: 5.9 Placebo: 9.5 HR: 0.57 (0.34-0.95); p=0.03	Disability progression confirmed at 24 week: Ocrelizumab: 7.9 Placebo: 11.5 HR: 0.63 (0.40-0.98); p=0.04	Confirmed disability progression for ≥12 weeks: Ocrelizumab: 32.9 Placebo: 39.3 HR: 0.76 (0.59-0.98); p=0.03 Confirmed disability progression for ≥24 week: Ocrelizumab: 29.6 Placebo: 35.7 HR: 0.75 (0.58-0.98); p=0.04	Disability worsening confirmed at 3 months (pooled analysis): Ofatumumab: 10.9 Teriflunomide: 15.0 HR: 0.66 (0.50 to 0.86) p=0.002 Disability worsening confirmed at 6 months (pooled analysis): Ofatumumab: 8.1 Teriflunomide: 12.0 HR: 0.68 (0.50 to 0.92); p=0.01		Worsening of disability confirmed at 12 weeks (pooled analysis): Ublituximab: 5.2 Teriflunomide: 5.9 HR: 0.84 (0.50 to 1.41); p=0.51	
**Mean percent change in brain volume (95% CI)**	Brain volume change from baseline to week 96 Placebo: -9.9 (37.0) vs Rituximab: -10.8 (40.3) p=0.62	n/a	Brain-volume change from week 24 to 96: Ocrelizumab: -0.57 (-0.66 to -0.49) Placebo: -0.74 (-0.83 to -0.65) Difference (%): 22.8; p=0.004	Brain-volume change from week 24 to 96: Ocrelizumab: -0.64 (-0.73 to -0.54) Placebo: -0.75 (-0.85 to -0.65) Difference (%): 14.9; p=0.0	Mean percent change in brain volume from week 24 to 120: Ocrelizumab: –0.90 (–1.00 to –0.80) Placebo: –1.09 (–1.24 to –0.95) HR: 17.5 (3.2 to 29.3); p=0.02	Annual rate of change: Ofatumumab: −0.28 (−0.34 to −0.22) Teriflunomide: −0.35 (−0.41 to −0.29) HR: 0.07 (−0.02 to 0.15); p=0.12	Annual rate of change: Ofatumumab: −0.29 (−0.35 to −0.23) Teriflunomide: −0.35 (−0.42 to −0.29) HR: 0.07 (−0.02 to 0.15); p=0.13	Percent change in brain volume from baseline to week 96: Ublituximab: −0.20 (−0.23 to −0.17) Teriflunomide: −0.13 (−0.16 to −0.10) Difference: −0.07 (−0.11 to −0.04)	Percent change in brain volume from baseline to week 96: Ublituximab: −0.19 (−0.23 to −0.16) Teriflunomide: −0.18 (−0.21 to −0.15) Difference: −0.02 (−0.05 to 0.02)

n/a, not applicable.

#### 4.1.2 Efficacy of rituximab in PPMS

The phase II/III OLYMPUS trial evaluated the efficacy and safety of rituximab in 439 patients with PPMS (64). In this study, 292 patients received rituximab at weeks 0, 2, 24, 26, 48, 50, 72, and 74, and were compared to patients who received placebo (**Table 3**). Although time to confirmed disease progression (CDP) was not significantly different between groups, T2-weighed lesion volume from baseline to week 96 increased less in patients who received rituximab.

Consistent with these data, retrospective studies have reported a significant reduction of disease activity, with reduced ARR, T2, and gadolinium-enhancing lesions, in patients treated with rituximab (90, 91).

### 4.2 Ocrelizumab

#### 4.2.1 Efficacy of ocrelizumab in RMS

##### 4.2.1.1 Phase II

Ocrelizumab efficacy has been studied in patients with RRMS in a phase II placebo-controlled clinical trial (92) (**Table 2**). Among the 220 patients included, 204 (93%) completed the 24-week study, and 218 (99%) were included in the intention-to-treat analysis. At week 24, the number of gadolinium-enhancing T1 lesions was reduced in patients treated with 600 mg (89% reduction) and 2000 mg (96% reduction) ocrelizumab compared to those in the placebo group. The ARR showed 80% and 73% reductions in the 600 mg and 2000 mg ocrelizumab groups, respectively, compared to the placebo group.

##### 4.2.1.2 Phase III

Phase II results have been confirmed in two phase III trials—OPERA I and OPERA II (4) (**Table 3**). At the end of the 96-week treatment period, 86.3% and 89.3% of patients who received ocrelizumab, and 76.6% and 82.7% of those who were given IFN-β-1a, had completed the study (for OPERA I and OPERA II, respectively). The ARR was lower with ocrelizumab compared to IFN-β-1a at 96 weeks (0.16 *vs*. 0.29), corresponding to 46% and 47% reductions in OPERA I and OPERA II, respectively. A relative risk reduction of 40% was observed for both 12-week and 24-week CDP with ocrelizumab compared to IFN-β-1a (9.1% and 6.9% for ocrelizumab; 13.6% and 10.5% for IFN-β-1a). The total mean number of gadolinium-enhancing T1 lesions was lower with ocrelizumab than with IFN-β-1a (94% fewer lesions with ocrelizumab in OPERA I and 95% in OPERA II). Ocrelizumab reduced the total number of Gd-enhancing T1 lesions by 94% in OPERA I and 95% in OPERA II) and the total number of new or newly enlarged T2 lesions by 77% in OPERA I and 83% in OPERA II. Finally, the percentage of patients who achieved no evidence of disease activity (NEDA3) was higher with ocrelizumab compared to IFN-β-1a (64–89% difference), and the brain volume loss was reduced by 14.9% to 22.8% in the ocrelizumab group. However, these results were inconclusive due to a failure of the statistical hierarchical testing procedure.

In the open-label extension study, patients switching from IFN-β-1a to ocrelizumab (at two years) had a similar ARR at five years (93). The proportion of patients with CDP at five years was lower with ocrelizumab compared to patients switching from IFN-β-1a to ocrelizumab (16.1% and 21.3%, respectively), but the risk of CDP after switching was similar. MRI activity (total number of T1 Gd-enhancing lesions and new/enlarging T2 lesions) was lower in patients switching from IFN-β-1a to ocrelizumab, with similar results compared to patients treated with ocrelizumab. Brain volume loss was unchanged in patients treated with ocrelizumab for five years.

#### 4.2.2 Efficacy of ocrelizumab in PPMS

##### 4.2.2.1 Phase III

The placebo-controlled ORATORIO trial evaluated the efficacy of ocrelizumab in patients with PPMS (5) (**Table 3**). Overall, 82% of patients who received ocrelizumab and 71% of those in the placebo group completed the 120-week treatment period. A relative risk reduction of 23% was reported for 12-week CDP with ocrelizumab compared to placebo (32.9% *vs*. 39.3%). This result was confirmed for 24-week CDP, with a relative risk reduction of 25%. Ocrelizumab also induced a 29.3% reduction in the timed 25-foot walk performance from baseline to week 120 compared to placebo. However, the SF-36 Physical Component Summary score was unchanged between the two groups. At 120 weeks, the total volume of T2 lesions, the number of new or enlarging T2 lesions, and the brain volume loss were reduced in ocrelizumab-treated patients. However, a bias in favor of the ocrelizumab group was introduced because there was more withdrawal in the placebo group than in the ocrelizumab group (34% and 21%, respectively), and this was considered to indicate a greater disease progression. When censoring was applied at withdrawal to these patients, the effect of ocrelizumab decreased, with an odds ratio of 0.86 (95% confidence interval: 0.62–1.19).

### 4.3 Ofatumumab

#### 4.3.1 Efficacy of ofatumumab in RMS

##### 4.3.1.1 Phase II

Ofatumumab was first evaluated in a placebo-controlled phase II trial involving 38 patients with RRMS (94). Patients received two IV infusions of ofatumumab at three different doses. Significant reductions in the number of T1 Gd-enhancing lesions, new T1 Gd-enhancing, and new and/or enlarging T2 lesions were observed in ofatumumab-treated patients (**Table 2**).

The MIRROR phase IIb trial evaluated the efficacy of SC administration of ofatumumab *vs*. placebo (68). In this 48-week study, patients received ofatumumab 3 mg, 30 mg, or 60 mg every 12 weeks, ofatumumab 60 mg every four weeks, or a placebo. Patients in the placebo group were given 3 mg ofatumumab at week 12. The cumulative number of new lesions was reduced by 65% for all ofatumumab dose groups compared to the placebo group from weeks 0 to 12. From weeks 4 to 12, the mean rate of new Gd-enhancing lesions was reduced, from 71% to 92% across ofatumumab groups. A ≥ 90% suppression of new lesions was observed over 12 weeks with cumulative doses ≥ 30 mg and 9% to 22% of patients across the ofatumumab groups relapsed *vs*. 25% in the placebo group (**Table 2**). Patients who received ofatumumab also experienced fewer relapses (9% to 22% across groups) than those in the placebo group over the 24-week treatment period.

##### 4.3.1.2 Phase III

The ASCLEPIOS I and II trials evaluated the efficacy of ofatumumab in patients with RMS (6). In ASCLEPIOS I, patients who received ofatumumab had a lower adjusted ARR (0.11) compared to those in the teriflunomide group (0.22), corresponding to a 50.5% relative reduction. Similar results were obtained in ASCLEPIOS II with a 58.5% relative reduction (**Table 3**). The mean number of T1 gadolinium-enhancing lesions was decreased with ofatumumab (0.01) compared to with teriflunomide (0.45) in ASCLEPIOS I (97% relative reduction with ofatumumab). The corresponding values in ASCLEPIOS II were 0.03 for ofatumumab and 0.51 for teriflunomide, corresponding to a 94% relative reduction. The number of new or enlarging lesions on T2-weighted MRI was reduced in the ofatumumab group in both ASCLEPIOS I (82% relative reduction) and ASCLEPIOS II (85% relative reduction). Finally, the annualized rate of brain volume loss did not differ significantly between patients treated with ofatumumab and those treated with teriflunomide.

### 4.4 Ublituximab

#### 4.4.1 Ublituximab efficacy in RMS

##### 4.4.1.1 Phase II

Ublituximab has been designed to induce higher ADCC compared to other anti-CD20 mAbs (46). In the phase II placebo-controlled 48-week trial, 36 patients with RMS received three ublituximab infusions (150 mg on day 1, 450-600 mg on day 15 and week 24) (**Table 2**). No T1 gadolinium-enhancing lesions were detected at weeks 24 and 48 (p = 0.003) and the T2 lesions volume was decreased by 10.6% at week 48 (p = 0.002) in ublituximab-treated patients. The annualized relapse rate was low (0.07), with 93% of patients remaining relapse-free during the study.

##### 4.4.1.2 Phase III

The phase III ULTIMATE I and II trials evaluated the efficacy (**Table 3**) and safety of ublituximab in patients with RMS (7). At 96 weeks, ublituximab treatment resulted in a lower ARR than teriflunomide (0.08 *vs*. 0.19 in ULTIMATE I; 0.09 *vs*. 0.18 in ULTIMATE II). Fewer T1 Gd-enhancing lesions were detected with ublituximab compared to teriflunomide in both trials (0.02 *vs*. 0.49 in ULTIMATE I; 0.01 *vs*. 0.25 in ULTIMATE II). The total number of new or enlarging lesions was also reduced in ublituximab-treated patients compared to the teriflunomide group (0.21 *vs*. 2.79 in ULTIMATE I; 0.28 *vs*. 2.83 in ULTIMATE II). In the ULTIMATE I trial, 44.6% of ublituximab-treated patients showed no evidence of disease activity (*vs*. 15% in the teriflunomide group). Similar results were reported in the ULTIMATE II trial, with 43% of the ublituximab group showing no evidence of disease activity *vs*. 11.4% in the teriflunomide group. However, no significant difference in the worsening of disability at 12 weeks was found between ublituximab- and teriflunomide-treated patients in the pooled analysis of the two trials (5.2% *vs*. 5.9%). Finally, brain volume change was not significant between the two groups.

## 5 Safety of anti-CD20 mAbs

### 5.1 Adverse events after anti-CD20 therapy

#### 5.1.1 Infusion/injections-related reactions

Infusion/injections-related reactions (IRRs) are the most common adverse events reported in patients treated with anti-CD20 mAbs (**Table 4**). Administration-related reactions generally occur within 24 hours of the first injection/infusion and decrease with subsequent doses. In the phase II HERMES and phase II/III OLYMPUS trials, 67.1% to 78.3% of patients treated with rituximab experienced IRRs following the initial infusion (63, 64). This percentage decreased to 20.3% to 22.6% after the second infusion and 4.9% after the eighth infusion. IRRs were mild-to-moderate, with 7.4% of rituximab-treated patients presenting grade 3 (63) and no grade 4 adverse events reported (63, 64). In the RIFUND-MS trial, 40.9% of patients treated with rituximab reported infusion-related reactions. The OPERA I/II study reported a 34.3% incidence of IRRs for ocrelizumab *vs*. 9.7% for IFN-β-1a (4). In the phase III ORATORIO study, the incidence of IRR was 39.9% for ocrelizumab *vs*. 25.5% for placebo (5). The phase II MIRROR study revealed that IRRs were more frequent with higher doses of ofatumumab and a shorter administration time (68). In phase III ASCLEPIOS trials, 20.2% of patients who received ofatumumab experienced mild-to-moderate IRRs, predominantly during the first injection (99.8% of IRRs), vs. 15% in patients receiving a placebo in the teriflunomide group (6). In the phase II trial, ublituximab caused grade 1 or 2 IRRs (50% of patients), although 77% of ublituximab infusions did not induce IRRs (46). Comparable results were reported in ULTIMATE I/II trials, with an occurrence of IRRs in 47.7% of ublituximab-treated patients (7). The frequency of these adverse events was higher with the first dose and subsequently decreased, as reported with the other anti-CD20 mAbs.

**Table 4:** Most common adverse events observed with rituximab, ocrelizumab, ofatumumab and ublituximab in phase II/III trials.

	Rituximab			Ocrelizumab			Ofatumumab		Ublituximab	
	Phase II HERMES Hauser et al., 2008	Phase II/II OLYMPUS, Hawker et al., 2009	Phase III RIFUND-MS Svenningsson et al., 2022	Phase III OPERA I Hauser et al., 2017	Phase III OPERA II Hauser et al., 2017	Phase III ORATORIO Montalban et al., 2017	Phase III ASCLEPIOS I Hauser et al., 2020	Phase III ASCLEPIOS II Hauser et al., 2020	Phase III ULTIMATE I Steinman et al., 2022	Phase III ULTIMATE II Steinman et al., 2022
**Administration-related reactions** **(% patients)**	Week 0: Rituximab: 78.3% Placebo: 40.0% Week 2: Rituximab: 20.3% Placebo: 40.0%	Week 0: Rituximab: 67.1% Placebo: 23.1% Week 2: Rituximab: 22.6% Placebo: 15.1% Week 74: Rituximab: 4.9% Placebo: 7.2%	Rituximab: 40.9% DMF: n/a	Ocrelizumab: 0.9% IFNb-1a: 6.4%	Ocrelizumab: 37.6% IFNb-1a: 12.0%		Ofatumumab: 16.1% Teriflunomide:16.5%	Ofatumumab: 24.1% Teriflunomide: 13.5%	Ublituximab: 44.0% Teriflunomide: 6.9%	Ublituximab: 51.5% Teriflunomide: 17.6%
**Infections (% patients)** All events Serious adverse event	Rituximab: 69.6% Placebo: 71.4% Rituximab:2.9% Placebo: 5.7%	Rituximab: 68.2% Placebo: 65.3% Rituximab: 4.5% Placebo: <1.0%	Upper respiratory tract: Rituximab: 61.5% DMF: 59.9% Urinary tract: Rituximab: 8.6% DMF: 5.1% Rituximab: 0.8% DMF: 0.7%	Ocrelizumab: 56.9% IFNb-1a: 54.3% Ocrelizumab: 1.2% IFNb-1a: 2.9%	Ocrelizumab: 60.2% IFNb-1a: 52.5% Ocrelizumab: 1.4% IFNb-1a: 2.9%	Ocrelizumab: 71.4% Placebo: 69.9% Ocrelizumab: 6.2% Placebo: 5.9%	Ofatumumab: 49.2% Teriflunomide: 51.5% Ofatumumab: 2.6% Teriflunomide: 1.5%	Ofatumumab: 53.8% Teriflunomide: 53.8% Ofatumumab: 2.5% Teriflunomide: 2.1%	Ublituximab: 48.5% Teriflunomide: 48.4% Ublituximab: 5.5% Teriflunomide: 2.2%	Ublituximab: 62.1% Teriflunomide:60.4% Ublituximab: 4.4% Teriflunomide:3.7%
**Neoplasm**	1 malignant thyroid neoplasm	n/a	n/a	Ocrelizumab: 0.7% IFNb-1a: 0.2%	Ocrelizumab: 0.2% IFNb-1a: 0.2%	Ocrelizumab: 2.3% Placebo: 0.8%	Ofatumumab: 0.6% Teriflunomide: 0.6%	Ofatumumab: 0.4% Teriflunomide: 0.2%	Ublituximab: 0% Teriflunomide: 0%	Ublituximab: 0.7% Teriflunomide: 0.4%

n/a, not applicable.

Murine-chimeric antibodies (rituximab and ublituximab) are more likely to cause immunogenic reactions than humanized (ocrelizumab) and fully human (ofatumumab) mAbs (45, 48). Human anti-chimeric antibodies were detected in 24.1% of patients treated with IV rituximab in the HERMES trial and 7% (*vs*. 6.3% for patients who received the placebo) in the OLYMPUS trial (63, 64). In the OPERA trials, anti-drug and neutralizing antibodies were detected in 0.4% and 0.1% of ocrelizumab-treated patients, respectively (4). In ORATORIO, 1.9% and 0.2% of patients developed anti-drug and neutralizing antibodies, respectively (5). No neutralizing antibodies were detected after ofatumumab treatment, and 0.2% of patients developed anti-drug antibodies (6). Data show that levels of neutralizing antibodies are very low, and there is no robust proof of a clear pathogenicity induced by these antibodies.

The relatively lower CDC potency of ocrelizumab and ublituximab compared with rituximab and ofatumumab might also lower the IRR incidence. Premedication (corticoids, antihistamines, and/or antipyretics) is required/recommended prior to IV infusion of rituximab, ocrelizumab, and ublituximab to lessen the severity and/or reduce the number of these events. Ofatumumab IRRs should be reduced compared to other anti-CD20 mAbs because SC administration is thought to generate reduced injection-related adverse events compared with IV infusions.

#### 5.1.2 Hypogammaglobulinemia

Although anti-CD20 mAbs do not affect plasma cells, B cell-depleting therapies may lead to a decline in serum Igs, increased infection risk, and lower response to vaccines. While MS clinical trials of rituximab, ocrelizumab, and ofatumumab have shown there to be a stronger decrease in IgM levels than in IgG and IgA levels over time, short-term follow-up results have indicated that there is no association with an increased risk of infection for patients (4–6, 63, 64). Serum levels of Ig were below the LLN in 7.9% of rituximab-treated patients (*vs*. 3% for the placebo group) (63). While the levels of IgM were low in 22.4% of patients who received rituximab (*vs*. 8.6% for the placebo group), median levels of IgM, IgG, and IgA across all patients stayed above the LLN for the duration of the trial.

The phase III trials and extended studies reported an association between the rate of serious infections and the reduction of Ig levels after prolonged ocrelizumab exposure (4, 5, 93). For most patients, Ig levels stayed above the LNN over five years (93). The percentages of patients with a decrease below the LLN at year 5 were 5.4% for IgG, 5.1% for IgA, and 29.5% for IgM. In phase III ASCLEPIOS trial, the mean levels of IgG did not decrease below the LLN with monthly ofatumumab treatment over 3.5 years compared with baseline values (95). A decrease in the mean IgM value was observed with ofatumumab, but this was not associated with the risk of infections, including serious infections (96). A reduction in serum IgM levels was observed over time, but for most patients, the levels remained above the LLN. The proportion of patients with IgM levels below the LLN at any time during the post-baseline visit was higher among patients who received ofatumumab (17.7%) *vs*. teriflunomide (6.6%). The proportion of patients with IgG below the LLN at any time during the post-baseline visit was lower in patients who received ofatumumab (14.2%) *vs*. teriflunomide (22.9%). In addition, a baseline quartile analysis from the extension study demonstrated that while the mean IgM levels decreased over time, they were maintained above the LLN from baseline to week 68, while the mean IgG levels remained stable over time (95).

Since CD20 is not expressed on plasma cells, IgG levels and existing humoral protection are maintained after treatment with anti-CD20 mAbs. It is essential to monitor IgG levels before, during, and after treatment to minimize the risks of serious infections.

#### 5.1.3 Infections

As mentioned earlier, serum Ig decline may increase infection risk in anti-CD20-treated patients. Phase III clinical trials revealed a comparable incidence of infections between anti-CD20 mAbs and comparators. The most common infections reported with rituximab in the RIFUND-MS trial were upper respiratory tract, urinary tract, and sinusitis infections (65)(**Table 4**). Serious infections occurred in two (0.8%) patients treated with rituximab and one (0.7%) patient who received dimethyl fumarate.

The incidence of infection was 56.9% to 60.2% in the ocrelizumab group *vs*. 52.5% to 54.3% in the IFN-β-1a group in the OPERA I/II trial (4). The most common infections were upper respiratory tract infection, nasopharyngitis, and urinary tract infection. 5.9% of patients reported herpesvirus-associated infection in the ocrelizumab group (*vs*. 3.4% in the IFN-β-1a group). Serious infections were reported in 1.3% of ocrelizumab-treated patients *vs*. 2.9% of IFN-β-1a-treated patients. No opportunistic infections were recorded in any group (4). In the ORATORIO trial, the infection incidence was 71.4% in the ocrelizumab group *vs*. 69.9% in the placebo group (5). Upper respiratory tract infections and oral herpes infections were more frequent with ocrelizumab treatment than with placebo treatment (10.9% *vs*. 5.9%). The most common infections were nasopharyngitis, urinary tract infection, influenza, and upper respiratory tract infection. Serious infections occurred in 6.2% of patients treated with ocrelizumab *vs*. 5.9% treated with a placebo.

The percentage of patients with recorded infections was 51.6% in the ofatumumab group and 52.7% in the teriflunomide group in the ASCLEPIOS I/II trials (6). Similar results were obtained from the extension study, which reported an infection incidence of 54.3% in ofatumumab-treated patients after 3.5 years of treatment (95). The most common infections were nasopharyngitis, upper respiratory tract infection, and urinary tract infection. A herpesvirus-associated infection was reported in 4.9% of the ofatumumab group and 4.2% of the teriflunomide group (6). Serious infections occurred in 2.5% of patients in the ofatumumab group *vs*. 1.8% in the teriflunomide group (6). Faster B cell repletion and persistence of splenic B cells with SC treatment may decrease the risk of infections.

In the ULTIMATE I/II clinical trials, infections occurred in 55.8% of patients treated with ublituximab *vs*. 54.4% of patients who received teriflunomide. Serious infections were reported in 5% and 4.4% of ublituximab-treated patients *vs*. 2.9% of teriflunomide-treated patients (7). Grades 1 or 2 herpesvirus-associated infections were reported in 5.7% of patients in the ublituximab group *vs*. 4.6% in the teriflunomide group. The most frequent infections were related to the respiratory tract. No opportunistic infections were reported.

#### 5.1.4 Risk of SARS-CoV-2 infection

Anti-CD20 therapies may expose patients with MS to SARS-CoV-2 infections. Evidence has shown that patients with MS present incidence, risk factors, and outcomes for SARS-CoV-2 that are similar to those of the general population (97–100). Risk factors for severe forms of SARS-CoV-2 include neurological disability, an older age, and comorbidities. A multi-center retrospective study conducted in France found no association between hospitalization for SARS-CoV-2 and anti-CD20 therapies (98). Most ocrelizumab-treated patients infected with severe SARS-CoV-2 developed a mild-to-moderate disease course without hospitalization. Recent work also reported SARS-CoV-2 infections of mostly mild-to-moderate severity in 245 ofatumumab-treated patients. Most patients recovered from SARS-CoV-2 infection without hospitalization or treatment discontinuation (101). However, an Italian cohort study in 844 patients with MS with SARS-CoV-2 infection reported an increased risk of severe SARS-CoV-2 in people treated with ocrelizumab or rituximab compared to untreated individuals (99). There was also a trend towards worse clinical outcomes with a longer duration of anti-CD20 therapies compared to other therapies. Patients treated with an anti-CD20 therapy for longer might be at a higher risk of SARS-CoV-2 due to decreased antibody responses (100).

#### 5.1.5 Progressive multifocal leukoencephalopathy

Rare cases of progressive multifocal leukoencephalopathy (PML) caused by the John Cunningham virus have occurred in patients treated with anti-CD20 antibodies. Notably, no PML cases have been reported in those receiving rituximab treatment for MS (63–65). Ten cases of PML have been recorded in those receiving ocrelizumab for MS (102). However, 9 of these cases previously received natalizumab or fingolimod and were considered carry-over cases. Another study reported a case of PML in a 78-year-old patient with PPMS who was treated with ocrelizumab for two years (103). In this case, the patient did not receive previous disease-modifying therapy. However, his age and the presence of lymphopenia increased the risk of developing PML. No reported cases of PML associated with ofatumumab or ublituximab in patients with MS (6, 95). Taken together, these findings indicate a minimal risk of PML under anti-CD20 treatment for MS. However, long-term safety data are missing, and vigilance is necessary for the elderly or patients switching from other disease-modifying therapies.

#### 5.1.6 Neoplasms

Neoplasms occurring after anti-CD20 therapy are rare events. Only one patient presented with a thyroid neoplasm after rituximab therapy in the HERMES trial (63). Furthermore, the incidence of neoplasms in patients with MS was similar to that of the general population in a retrospective study that evaluated cancer risk with disease-modifying therapies (104). Furthermore, long-term analysis from the rituximab global safety database indicated no increased risk of malignancy (rate of 4.2 per 1 000 patients) in patients with rheumatoid arthritis who received rituximab (105). Similar results were obtained from the Rheumatoid Arthritis Global Clinical Trial Program (106). Prolonged exposure to rituximab over eleven years did not increase the rate of neoplasms in these patients. A higher incidence of neoplasms (particularly in the breast) was found with ocrelizumab treatment in RMS and PPMS trials compared to comparator and placebo groups (4, 5). Neoplasms occurred in 0.5% of patients treated with ocrelizumab, in 0.2% of those treated with IFN-β-1a in the OPERA I/II trials, and in 2.3% of ocrelizumab-treated patients compared to 0.8% in the placebo group in the ORATORIO study. The incidence of neoplasms was comparable to that of the general population, but there was an imbalance in the occurrence of breast cancer, which should be further investigated. In the MIRROR study, one case of malignant melanoma was reported in the 60 mg ofatumumab group (68). In the ASCLEPIOS trials, neoplasms were reported in 0.5% of patients in the ofatumumab group and 0.4% in the teriflunomide group (6). Similarly, only rare cases of neoplasm occurred in ublituximab-treated patients (0.7%) compared to patients who received teriflunomide (0.4%) (7).

#### 5.1.7 Late-onset neutropenia

Several studies have suggested that rituximab can cause late-onset neutropenia (LON). LON, defined as grade III-IV neutropenia, occurs at least 3-4 weeks after the last rituximab infusion and has no other identified cause (107–112). The OPERA I/II trials reported an incidence of mild neutropenia in 14.7% of patients treated with ocrelizumab *vs*. 40.9% of patients treated with IFN-β-1a (4). The ORATORIO trial reported an incidence of 13% in patients treated with ocrelizumab *vs*. 10% for those who received the placebo (5). Less than 1% of all patients exhibited grade 3 or 4 neutropenia. Only rare case reports have described LON in patients treated with ocrelizumab after the first infusion (113–115). Several mechanisms have been proposed to account for LON (112, 116–119): cell maturation arrest, potentially arising from defects in SDF-1 and preventing mature neutrophils from reaching the blood; IgG FcɣRIIIa158V/F polymorphism that leads to antibody-mediated elimination of neutrophils: or increase in BAFF levels during B cell.

Considering that LON is infrequent, not predictable, varies in terms of its onset and duration, and rarely causes significant infection, a blood count can be performed in the event of fever or infection in the months following anti-CD20 mAbs treatment.

### 5.2 Vaccination efficacy

Vaccination is critical in MS management because infections can aggravate MS symptoms. Many studies have evaluated the responses to vaccination in rituximab-treated patients and have shown reduced humoral responses, depending on the vaccines (120–124). The VELOCE study evaluated responses to selected vaccines in ocrelizumab-treated patients with RMS (125). The data indicated that ocrelizumab-treated patients had attenuated humoral responses to the tetanus toxoid vaccine, pneumovax, and the neo-antigens KHL vaccine. A recent study examined the hepatitis B seroconversion rate in 153 patients with MS who were receiving disease-modifying treatment (126). A switch to an anti-CD20 therapy during the series of vaccinations led to a decline in the seroprotection rate, from 66.7% at baseline to 18.2% to 52.2%. The seroprotection rate was higher in patients who had completed more of the scheduled vaccine doses before receiving anti-CD20 therapy. The decline in the seroconversion rate appeared to be relatively specific to anti-CD20 mAbs compared to other monoclonal antibodies like natalizumab.These studies indicate that vaccination should be carefully planned in patients treated with anti-CD20 mAbs. Patients should ideally complete their immunization at least four weeks prior to initiation of rituximab (127) and six weeks prior to initiation of ocrelizumab (67). Live vaccines should not be administrated until treatment cessation and B cell recovery (67). Ocrelizumab-treated patients should also receive inactivated seasonal influenza vaccines (67). The existing recommendations are to administer live attenuated vaccines (e.g., for chickenpox or measles) four weeks prior to the initiation of ofatumumab, and non-live and inactivated vaccines at least two weeks prior to treatment initiation (70).

#### 5.2.1 A focus on SARS-CoV-2 vaccination

Many studies have investigated the cellular and humoral responses following SARS-CoV-2 vaccination in patients with MS. Treatment with anti-CD20 mAbs has been found to significantly reduce spike-specific and receptor-binding domain-specific antibody and memory B cell responses in most patients, and this effect is ameliorated with longer durations from the last treatment and the extent of B cell reconstitution (128). Although most patients showed poor antibody responses to the vaccine, all patients generated robust CD4 and CD8 T cell responses. Consistent with these data, another study reported that patients treated with ocrelizumab had a lower serology response than untreated patients and healthy controls but showed a preserved T cell response to the SARS-CoV-2 vaccine compared with healthy controls (129). Achiron and colleagues also stated that despite a normal absolute lymphocyte count, only 22.7% of treated patients with ocrelizumab developed a humoral IgG response (130). These results were confirmed in a large cohort study of 1339 patients with MS (treated with a disease-modifying therapy or untreated) who received two doses of a SARS-CoV-2 mRNA vaccine (131). Multivariate analysis revealed decreased antibody levels in patients treated with ocrelizumab (231-fold decrease) and rituximab (20-fold decrease) compared with untreated patients. Higher antibody levels were detected when the time to the last administration was longer. A large cohort study (555 patients with MS) characterized the occurrence of immediate relapses following SARS-CoV-2 vaccination (132). The results indicated that the risk of relapse was not changed after the first or the second vaccine doses (median follow-up of 20 days and 38 days). The proportion of patients experiencing acute relapses was similar to that of non-vaccinated patients. Finally, several real-world evidence studies aimed at evaluating vaccine efficacy comparing SARS-CoV-2 infection severity in vaccinated and non-vaccinated patients with MS. An Italian study did not reveal any significant reduction in the hospitalization rate of vaccinated ocrelizumab-treated patients (16.7% post-vaccination *vs*. 19.4% pre-vaccination) (133). However, results from the Italian CovaXiMS (Covid-19 vaccine in Multiple Sclerosis) study indicated a significant reduction (70%) in the rate of hospitalization after SARS-CoV-2 vaccination, including in patients treated with ocrelizumab (134). This correlated with a small cohort showing that SARS-CoV-2 infections were more severe in non-vaccinated (33.3%) *vs*. vaccinated patients (25%) (135).

## 6 Anti-CD20 and pregnancy

Pregnancy poses challenges for the management of MS, which is typically diagnosed between the ages of 20 and 40 years. Historically, clinicians discouraged women with MS from having children, but the PRIMS study challenged this view (136). Indeed, no significant difference in the annualized relapse rate was found when comparing the pregnancy with the pre-pregnancy year. Anti-CD20 mAbs induce prolonged B cell depletion and can cross the placenta from the second trimester. A recent observational study evaluated the risk of disease reactivation during pregnancy and in the post-partum period following rituximab discontinuation (137). The relapse rate remained the same in the post-partum period compared with the pre-pregnancy period in women who suspended rituximab treatment within 6 months before conception. These results suggested that rituximab might exert long-lasting effects on MS disease activity. Disease activity was also analyzed in a cohort of women treated with rituximab or ocrelizumab 12 months before or during pregnancy (138). All women with MS exposed to rituximab/ocrelizumab before the last menstrual period were relapse-free during pregnancy, and 17.2% experienced a relapse post-partum.

Despite counseling strategies, an unplanned pregnancy can occasionally occur during or after anti-CD20 treatment. Several studies have assessed the risk of maternal rituximab exposure for the fetus. The largest study evaluated 231 pregnancies associated with maternal rituximab exposure in lymphoma or autoimmune diseases (139). Of 153 pregnancies with known outcomes (including two patients with MS), nearly 60% resulted in live births, with 24% of preterm neonates and only 2.2% of neonates with congenital malformations. The preterm birth rates in this group were higher than in the general population (10% to 12%), but they were comparable to rates observed in women with chronic conditions (140, 141). One case series evaluated 11 pregnancies in women with MS and neuromyelitis optica spectrum disorder who received rituximab within six months of conception (142). None of the women experienced relapse before conception or during pregnancy, and all children were healthy at birth. However, larger sample size is required to evaluate the safety of rituximab before, and during pregnancy in women with MS. Although these findings are reassuring, the FDA and EMA both recommend that women use contraception during rituximab treatment and avoid pregnancy for 12 months after the last rituximab exposure.

Limited information is available on the use of ocrelizumab during pregnancy. Pregnancy outcomes were reported in women with MS, rheumatoid arthritis and systemic lupus treated with ocrelizumab (20 mg to 2000 mg) (143). A total of 362 pregnancies were reported, including 267 in women with MS. Preliminary outcomes did not suggest an increased risk of adverse pregnancy outcomes, including spontaneous abortions or malformations. Pregnancy outcomes analyzed in a cohort of women treated with rituximab or ocrelizumab 12 months before or during pregnancy indicated that most pregnancies resulted in live births (88.2%) and there were few congenital anomalies (3.2% of live births) (138). Despite exposure six months before the last menstrual period or during pregnancy, most newborns had normal B cell counts. Another large study (1223 pregnancies) reported pregnancy outcomes in women with MS who received ocrelizumab (144). 70.7% of the 604 pregnancies with known outcomes resulted in live births (11% preterm; 1.6% with major congenital malformations). There were 2.2% ectopic pregnancies, 8.9% elective abortions/therapeutic abortions, 17.4% spontaneous abortions, and 0.8% stillbirths. Of the 236 pregnancies with fetal exposure, 72.5% resulted in live births, 0.8% were ectopic pregnancies, 12.7% elective abortions/therapeutic abortions, 12.3% spontaneous abortions, and 1.7% stillbirths. Consistent with previous findings, there is no evidence of an increased risk of congenital anomalies with ocrelizumab. According to current FDA et EMA recommendations, ocrelizumab should be avoided during pregnancy, and women should consider pregnancy 6 to 12 months (US *vs*. Europe) after the last infusion. This delay could be reduced to 2 to 3 months for women with active disease as monoclonal antibodies do not cross the placental barrier during the first trimester (145).

Data on the use of ofatumumab during pregnancy are also limited. According to animal studies, ofatumumab can cross the placental barrier and cause the depletion of B cells in the fetus (70). In the ALITHIOS trial, fourteen pregnancies were reported in patients exposed to ofatumumab during the first trimester. Of these pregnancies, three resulted in live birth; all were full-term deliveries with no birth defects, congenital anomalies, or serious infections for the newborns. Four pregnancies were ongoing at the data collection cut-off, and six resulted in early termination (95). Long-term safety data are awaited to confirm these reassuring data.

IgGs are large molecules with low expected breast milk transfer after the colostrum phase. Rituximab concentration was evaluated in mature breast milk from women who received 500 mg or 1 000 mg IV rituximab while breastfeeding. Results indicated that the relative infant dose was 0.08%, well below acceptable levels of 10% (146). Another small-size study confirmed that rituximab concentrations in breast milk and infant’s serum were low, suggesting a minimal transfer to the milk and no absorption of rituximab by breastfed infants (147). There is no published data on the potential effect of ocrelizumab, ofatumumab, and ublituximab in nursing mothers. The available data suggest that treatment with anti-CD20 mAbs during lactation might be safe for breastfed infants, yet it is not generally recommended by clinicians.

## 7 Conclusion

Anti-CD20 mAbs have emerged as an essential treatment option for patients with relapsing MS. Clinicians must be aware that anti-CD20 mAbs differ in their structures, target epitopes, dosing regimens, route of administration, and mechanisms of B cell depletion. All four antibodies have proven efficacious in controlling disease activity and disability progression. Long-term safety data from extended trials and real-life studies are starting to emerge, and it will be interesting to see how long-term B cell depletion affects Ig levels and infection risk. Furthermore, it would be worthwhile to understand whether anti-CD20 mAbs could be used as induction therapy before switching to a less potent immunosuppressive drug. Similarly, sequential therapy or spacing interval (for example, one year for IV administered anti-CD20 or every two months for SC injections) should be evaluated, in particular in stabilized patients.

## Author’s note

Authors had full control of the content and made the final decision for all aspects of this article.

## Author contributions

All authors conceptualized, reviewed and edited the manuscript for intellectual content. All authors contributed to the article and approved the submitted version.
